# Elevated Levels of Asymmetric Dimethylarginine (ADMA) in the Pericardial Fluid of Cardiac Patients Correlate with Cardiac Hypertrophy

**DOI:** 10.1371/journal.pone.0135498

**Published:** 2015-08-27

**Authors:** Zoltan Nemeth, Attila Cziraki, Sandor Szabados, Bernadett Biri, Sandor Keki, Akos Koller

**Affiliations:** 1 Department of Pathophysiology and Gerontology and Szentagothai Research Centre, University of Pecs, Medical School, Pecs, Hungary; 2 Heart Institute, University of Pecs, Medical School, Pecs, Hungary; 3 Department of Applied Chemistry, University of Debrecen, Faculty of Science, Debrecen, Hungary; 4 Institute of Natural Sciences, University of Physical Education, Budapest, Hungary; 5 Department of Physiology, New York Medical College, Valhalla, New York, United States of America; Goethe University, GERMANY

## Abstract

**Background:**

Pericardial fluid (PF) contains several biologically active substances, which may provide information regarding the cardiac conditions. Nitric oxide (NO) has been implicated in cardiac remodeling. We hypothesized that L-arginine (L-Arg) precursor of NO-synthase (NOS) and asymmetric dimethylarginine (ADMA), an inhibitor of NOS, are present in PF of cardiac patients and their altered levels may contribute to altered cardiac morphology.

**Methods:**

L-Arg and ADMA concentrations in plasma and PF, and echocardiographic parameters of patients undergoing coronary artery bypass graft (CABG, n = 28) or valve replacement (VR, n = 25) were determined.

**Results:**

We have found LV hypertrophy in 35.7% of CABG, and 80% of VR patients. In all groups, plasma and PF L-Arg levels were higher than that of ADMA. Plasma L-Arg level was higher in CABG than VR (75.7±4.6 μmol/L vs. 58.1±4.9 μmol/L, p = 0.011), whereas PF ADMA level was higher in VR than CABG (0.9±0.0 μmol/L vs. 0.7±0.0 μmol/L, p = 0.009). L-Arg/ADMA ratio was lower in the VR than CABG (VR_plasma_: 76.1±6.6 vs. CABG_plasma_: 125.4±10.7, p = 0.004; VR_PF_: 81.7±4.8 vs. CABG_PF_: 110.4±7.2, p = 0.009). There was a positive correlation between plasma L-Arg and ADMA in CABG (r = 0.539, p = 0.015); and plasma and PF L-Arg in CABG (r = 0.357, p = 0.031); and plasma and PF ADMA in VR (r = 0.529, p = 0.003); and PF L-Arg and ADMA in both CABG and VR (CABG: r = 0.468, p = 0.006; VR: r = 0.371, p = 0.034). The following echocardiographic parameters were higher in VR compared to CABG: interventricular septum (14.7±0.5 mm vs. 11.9±0.4 mm, p = 0.000); posterior wall thickness (12.6±0.3 mm vs. 11.5±0.2 mm, p = 0.000); left ventricular (LV) mass (318.6±23.5 g vs. 234.6±12.3 g, p = 0.007); right ventricular (RV) (33.9±0.9 cm^2^ vs. 29.7±0.7 cm^2^, p = 0.004); right atrial (18.6±1.0 cm^2^ vs. 15.4±0.6 cm^2^, p = 0.020); left atrial (19.8±1.0 cm^2^ vs. 16.9±0.6 cm^2^, p = 0.033) areas. There was a positive correlation between plasma ADMA and RV area (r = 0.453, p = 0.011); PF ADMA and end-diastolic (r = 0.434, p = 0.015) and systolic diameter of LV (r = 0.487, p = 0.007); and negative correlation between PF ADMA and LV ejection fraction (r = -0.445, p = 0.013) in VR.

**Conclusion:**

We suggest that elevated levels of ADMA in the PF of patients indicate upregulated RAS and reduced bioavailability of NO, which can contribute to the development of cardiac hypertrophy and remodeling.

## Introduction

The pericardium is a fluid filled double-layered sac that surrounds the heart and the proximal ends of the large coronaries. The space between the two layers is filled with serous fluid, called pericardial fluid (PF). One of the main physiological roles of the PF is providing a proper friction within the pericardium by lubricating the epicardial surface making possible the continuous movement of the heart in every beat [1]. For many years PF was considered as a passive ultrafiltrate of the plasma produced by hydrostatic pressure difference and osmotic concentration gradient between the plasma and the PF [2]. However, other studies using rabbits and dogs extended this simplistic view by further analyzing the composition of the PF [3].

One of the first extensive studies obtained detailed information regarding the composition of PF of 30 patients undergoing elective open heart surgery, and found that concentrations of small molecules (such as urea, uric acid, glucose and electrolytes) were essentially the same in both the PF and the plasma [4]. However, production of PF involves not only filtration processes, but also active mechanisms resulting in the accumulation of several biologically important substances, which are produced by the myocardium. Such substances are endothelins (ETs) [5], adenine nucleosides [6], angiotensin [7]. It has also been revealed that these substances present in higher concentration in the PF compared to the plasma. An increased concentration of these substances has been reported during cardiac diseases [8]. In addition, the composition of PF is altered in various cardiac diseases [9] and in cardiac hypertrophy [10]. Nevertheless, still relatively few studies are extant, which investigated the biochemical composition of PF to humans [11].

Nitric oxide (NO) is a multirole molecule, among others modulating vasomotor tone, and attenuating tissue proliferation and growth [12]. Several studies have demonstrated the anti-hypertrophic role of NO on cardiac muscle [13–15]. For instance, induced hypertrophied cardiomyocytes were inhibited by administration of NO [16]. Also, reduced NO availability has been associated with cardiac hypertrophy [17]. Previous studies established that asymmetric dimethylarginine (ADMA), being a false substrate limits the activity of endothelial nitric oxide (NO) synthase thus production of NO. In addition, our previous studies and others revealed that elevated levels of ADMA elicits the release of reactive oxygen species (ROS) by activation of the renin angiotensin system (RAS) leading to vascular dysfunction [18–20]. ADMA can accumulate due to enhanced production or reduced catabolism and excretion [21]. ADMA is produced by protein arginine methyltransferase type I (PRMT-1) via methylation from L-arginine (L-Arg) and mainly metabolized by dimethylarginine dimethyl-aminohydrolase (DDAH) [22]. Furthermore, ADMA has been identified as a risk factor for endothelial dysfunction and was shown to accelerating the progression of several cardiovascular diseases [23]. Thus, ADMA may serve as a biomarker indicating reduced bioavailability of NO [18–20]. Previous studies have shown that ADMA is partly excreted by the kidneys, and suggested that low urinary ADMA level predicts impaired cardiac function [24]. It has been reported by several studies that plasma levels of ADMA increase in chronic kidney disease (CKD) [25]. First, Vallance et al have shown a relationship in patients with chronic renal failure [25]. In addition, studies have shown that elevated plasma ADMA levels are implicated with the progression of chronic kidney disease (CKD) and increased the risk for cardiovascular disease [26, 27]. Pecchini et al compared findings of ELISA and LC-MS/MS methods regarding the measured level of ADMA, and found that from the same sample ADMA values were higher measured by ELISA compared to LC-MS/MS, however they concluded that ELISA overestimates ADMA concentration [28]. Also, Ronden et al showed that at increased concentration ADMA is excreted by the kidneys, however when GFR declines its excretion is lower. Also, in the presence of CKD the activity of PRMT increases with the consequent elevation in ADMA production, which could be further increased by the decreased catabolism due to reduced DDAH enzyme activity [29].

Indeed, clinical studies showed an elevated plasma level of ADMA in aortic valve stenosis [30] and revealed that ADMA is a predictor of heart diseases, such as acute coronary events [31]. Many of these patients usually undergo coronary artery bypass graft (CABG) and valve replacement (VR) surgeries.

Based on the aforementioned, we hypothesized that the level of ADMA in the PF may be different in patients with CABG and VR, which could contribute to the morphological changes of the heart. Thus, we measured the concentrations of ADMA in the PF obtained from patients who underwent either coronary artery bypass graft or valve replacement surgery. Moreover, we measured the levels of ADMA, and L-Arg in plasma of all groups, and in PF of CABG and VR patients, and made comparisons between the different groups.

## Methods

### Study description and clinical characterization

In the present study, we have investigated 73 patients at the Heart Institute at the Medical School, University of Pecs, Hungary. This is a cross-sectional investigation of 28 patients undergoing coronary artery bypass graft (CABG) surgery, and 25 undergoing cardiothoracic surgery for valve replacement (VR). We measured peripheral blood plasma level of ADMA in 20 non-cardiac patients (NCP). Written informed consent was obtained from all individuals before participation in the study. The Ethics Committee of the Medical School of University of Pecs (RKEB-4123/2011) approved the investigation and consent documents. The investigation conforms to the principal outlined in the Declaration of Helsinki. Plasma was harvested from NCP, and both plasma and PF were collected from the patients after median sternotomy and collected into heparinized vacutainer tubes.

### Echocardiography

All patients underwent complete 2-D transthoracic echocardiography before and after surgery. Two-dimensional (2-D), M-mode and Doppler echocardiography with automated border detection were performed using Hewlett-Packard Sonos 5500 echocardiograph with a 2.5 MHz transducer (Hewlett-Packard, USA). 2-D echocardiographic measurements were performed according to the recent European guidelines [32]. All echocardiographic measurements were performed by the same (blinded for the patients conditions) cardiologist having an expert license in transthoracic echocardiography, which minimized the variability and bias in the measurements. All the recorded images were analyzed off-line.

The following parameters were measured: left ventricular end-diastolic diameter (Dd), left ventricular end-systolic diameter (Ds), thickness of interventricular septum (IVS) and posterior wall (PW), right ventricular (RV), right atrial (RA), and left atrial (LA) area.

### Biochemical analysis

We have followed the procedure as previously described in detail [33]. PF and blood samples were centrifuged (3000 rpm, 30 min) immediately after collection. Supernatants were achieved at -75°C until biochemical analysis. L-Arg and asymmetric dimethylarginine (ADMA) were determined using liquid chromatography [34]. Quantification of ADMA and L-Arg derivatives was performed at the Department of Applied Chemistry, University of Debrecen. The sample preparation method and the chromatographic method were validated. Among other parameters, the robustness of the chromatographic system, the recovery of the amino acids from the samples and the repeatability have been checked. System suitability was also checked regularly during the analysis of the samples. Solid phase extraction (SPE) of the analytes were performed according to the method of Nonaka et al. [34]: 250 μL of plasma sample was mixed with 700 μL of pH = 9.00 borate buffer and L-homoarginine hydrochloride (Sigma, HArg) was used as internal standard (50 μL of 1000 μmol/L solution). The resulting mixture was passed through OASIS MCX 3cc SPE cartridges at 750 mbar in a 12-column manifold (J. T. Baker). Thus, 11 samples and a standard solution were prepared parallel. The standard was used to check the system suitability parameters, e.g. chromatographic resolution. Washing of the SPE cartriges was done respectively by borate buffer, water, and methanol (Sigma). The analytes were eluted with a mixture of concentrated aqueous ammonia (Reanal), water, and methanol with a volume ratio of 10/40/50. The solvent was evaporated beginning under nitrogen atmosphere and finished in vacuum at 60°C. The dry residue was dissolved in 200 μL of ultrafiltered deionized water (Millipore, Milli-Q) and derivatized according to Molnar-Perl et al. The samples (200 μL) were mixed with 63 μL of reagent solution containing OPA (ortho-phthaldialdehyde from Fluka) and MPA (3-mercaptopropionic acid from Aldrich) and incubated at RT for 10 min then cooled down to 5°C. For HPLC analysis, derivatized samples of 10 μL were injected into a Waters 2695 Separations Module equipped with a thermostable autosampler (5°C) and a column module (35°C). Separation was achieved with a Waters Symmetry SB C18 (4.6 x 150 mm, 3.5 μm) column and detected by a Waters 2745 Fluorescent detector (Waters Milford, MA, USA). Gradient elution at a flow rate of 1 mL/min was applied during the analysis with two mobile phases: A (20 mmol (NH_4_)_2_CO_3_ in water, pH = 7.50 ± 0.05) and B (acetonitrile). The gradient program was as follows: 0–16 min: 91% A and 9% B, 16–17 min: linear change to 70% A and 30% B and hold this for 5 minutes, 22–23 min: linear change to 91% A, 9% B and hold this for 12 minutes. The last two phases were to wash and regenerate the column for the next sample. Arginine and homoarginine were detected at λex = 337 nm, λem = 520 nm, and λem = 454 nm was used for (ADMA) and symmetric dimethylarginine (SDMA).

### Calculations and Statistical analysis

Left ventricular mass (LVM) was calculated using the American Society of Echocardiography (ASE) convention: LV mass = 0.8 (1.04 ([LVIDD + PWTD + IVSTD]^3^- [LVIDD]^3^)) + 0.6 g [2]. The left ventricular ejection fraction (LVEF) as the index of global systolic function was calculated according to the Simpson formula [35]. GFR values were estimated using the six variable modifications of diet in renal disease (MDRD) equation from creatinine levels, sex, race, and age [36]. Serum creatinine was evaluated by the kinetic rate-blanked Jaffe compensated assay [37]. Results are expressed as mean±SEM. Statistical analyses were performed with Microsoft Excel and SPSS. Statistically significant differences were determined using the Student’s two-tailed unpaired t-test. The eGFR of each of the groups was compared using ANOVA on Ranks analysis. The Pearson’s correlation test and linear regression analysis were used to calculate correlations between levels of L-Arg and ADMA, and levels of ADMA and echocardiographic parameters. p < 0.05 was considered as statistically difference.

## Results and Discussion

### Characteristics of patients

Descriptive statistics of NCP and patients with CABG or VR surgery are summarized in Table 1, which shows the major demographic and clinical characteristics, as well as concomitant risk factors and medications of patients. The mean ages and gender of both CABG and VR patients were similar. 35.7% of CABG and 80% of VR patients demonstrated LV hypertrophy. In general, the CABG patients had hypertension and most of them had a history of previous acute myocardial infarction (AMI). The serum creatinine (sCr), and eGFR were similar in both CABG and VR patients, albeit both patient groups mean eGFR indicate CKD stage 3. Pre-operative medications of the patients were similar, however patients of the CABG were treated with higher dose of aspirin and statin before surgery compared to patients of the VR patients. In this study, 53 Caucasian patients underwent cardiothoracic surgery: 28 for CABG, and 25 for VR. CABG surgical interventions were as follows: x1 CABG-0; x2 CABG-3; x3 CABG-16; x4 CABG-8; x5 CABG-1. VR surgical interventions were as follows: AVR-17; MVR-7; AVR-MVR-1. The types of surgery are summarized in Table 2.

**Table 1:** Characteristics of the patients and medications.

Variable	NCP (n = 20)	CABG (n = 28)	VR (n = 25)	p
Pre-operative data				
Age (year)	42.0±3.5	59.7±1.5	56.4±4.1	0.331
Sex (male/female)	11/9	17/11	15/10	0.870
Hypertension*	-	27	18	0.013
Cardiac hypertrophy	-	0	19	0.001
Diabetes mellitus	-	9	5	0.326
Previous AMI	-	12	0	0.000
sCr (μmol/L)	74.0±5.4	78.8±6.9	75.0±3.6	0.649
Estimated GFR (ml/min/1.73m^2^)	-	58.54±1.3	58.64±0.8	0.947
**Pre-operative medication**		85% in combination/15% in monotherapy^#^	75% in combination/25% in monotherapy^#^	
Beta-blocker	-	23	18	0.809
Ca-channel blocker	-	8	5	0.671
ACE-inhibitor	-	14	16	0.129
AT-receptor blocker	-	4	1	0.208
Nitrate	-	0	0	0.000
Aspirin	-	21	4	0.000
Anti-diabetic	-	7	3	0.561
Statin	-	25	9	0.000
Diuretic	-	11	6	0.242

Data are mean **±** SEM.

*indicating blood pressure of 140/90 was considered normal in both cardiac groups [63].

^#^indicating medications in monotherapy for CABG (using beta-blocker or ACE inhibitor) and VR (using diuretic or ACE inhibitor).

CABG: coronary artery bypass graft; VR: valve replacement; AMI: acute myocardial infarction, Estimated GFR: estimated GFR calculated by the Modification of Diet in Renal Disease (MDRD) GFR, sCr: serum creatitine, NCP–non-cardiac patients; CABG–coronary artery bypass graft; VR–valve replacement.

**Table 2:** Types of surgery operation.

Operation	
CABGx1	0
CABGx2	3
CABGx3	16
CABGx4	8
CABGx5	1
AVR	17
MVR	7
AVR+MVR	1
Total	53

CABG: coronary artery bypass graft (the number indicates the vessels involved); VR: valve replacement; AMI: acute myocardial infarction.

### L-arginine and ADMA levels in NCP, CABG, and VR patients

**Fig 1:**
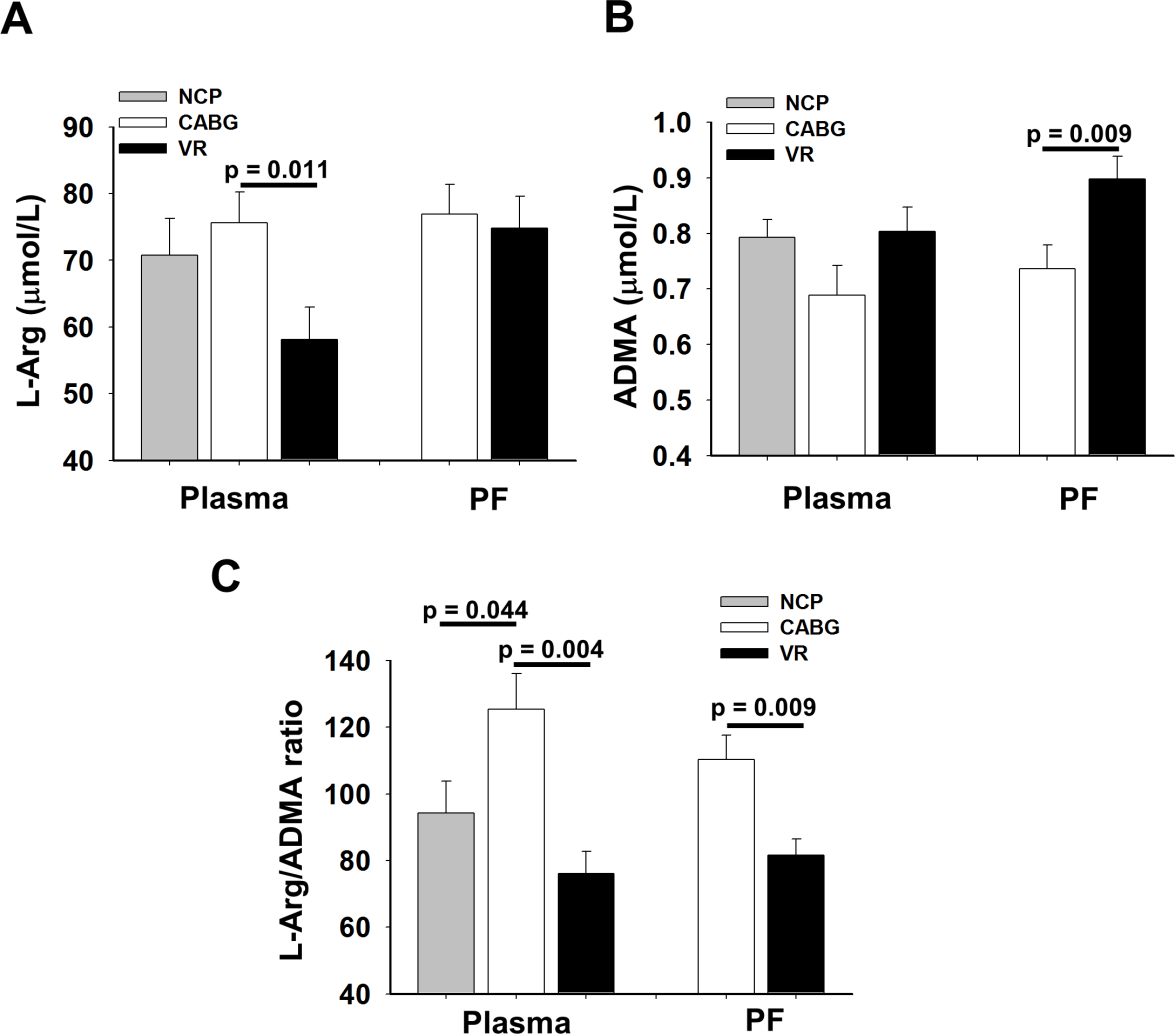
L-Arg and asymmetric dimethylarginine (ADMA) in plasma of non-cardiac patients (NCP) (n = 20), and in plasma and pericardial fluid (PF) of patients undergoing coronary artery bypass graft (CABG, n = 28) or valve replacement (VR, n = 25) surgery. (A) Levels of L-Arg in plasma and PF. (B) Levels of ADMA in plasma and PF. (C) ratios of L-Arg and ADMA in plasma and PF. Mean ±SEM. * indicates significant (p<0.05) differences between CABG and VR patients. Substrate availability indicated by the ratio, which is significantly increased in CABG compared to NCP and VR.

There was no significant difference in plasma levels of L-Arg and ADMA between the NCP, and the patients undergoing open-heart surgery (L-Arg_NCP_: 70.8±6.0 μmol/L vs. L-Arg_CABG_: 75.7±4.6 μmol/L, p = 0.513; L-Arg_NCP_: 70.8±6.0 μmol/L vs L-Arg_VR_: 58.1±4.9 μmol/L, p = 0.106; ADMA_NCP_: 0.8±0.0 μmol/L vs. ADMA_CABG_: 0.7±0.0 μmol/L, p = 0.144; ADMA_NCP_: 0.8±0.0 μmol/L vs. ADMA_VR_: 0.8±0.0 μmol/L, p = 1.707) (Fig 1A and 1B). In CABG patients, the plasma L-Arg levels were significantly higher compared to the VR patients (75.7±4.6 μmol/L vs. 58.1±4.9 μmol/L, p = 0.011), whereas there was no significant difference in pericardial fluid L-Arg levels between the CABG and the VR patients (76.9±4.4 μmol/L vs. 74.8±0.0 μmol/L, p = 0.748) (Fig 1A). VR patients exhibited significantly higher ADMA levels in PF than that of CABG group (0.9±0.0 μmol/L vs. 0.7±0.0 μmol/L, p = 0.009; Fig 1B). There was a significant difference in L-Arg/ADMA ratio in plasma between the NCP and CABG patients (94.2±9.5 vs. 125.4±10.7, p = 0.044), but not between NCP and VR patients (94.2±9.5 vs. 78.3±7.7, p = 0.197) (Fig 1C). Furthermore, the L-Arg/ADMA ratio both in plasma and PF was significantly higher in the CABG compared to the VR patients (in plasma: 125.4±10.7 vs. 76.1±6.6, p = 0.004, in PF: 110.4±7.2 vs. 81.7±4.8, p = 0.009; Fig 1C). We found a significant inverse correlation between plasma L-Arg and eGFR in the CABG group (r = -0.367, p = 0.027). We found no significant correlation between the L-Arg/ADMA ratio and eGFR neither in plasma nor in PF of VR patients (pl L-Arg/ADMA ratio vs. eGFR: r = 0.200, p = 0.169; PF L-Arg/ADMA ratio vs. eGFR: r = 0.073, p = 0.128).

### Correlation between the levels of L-Arg and ADMA in plasma and PF

**Fig 2:**
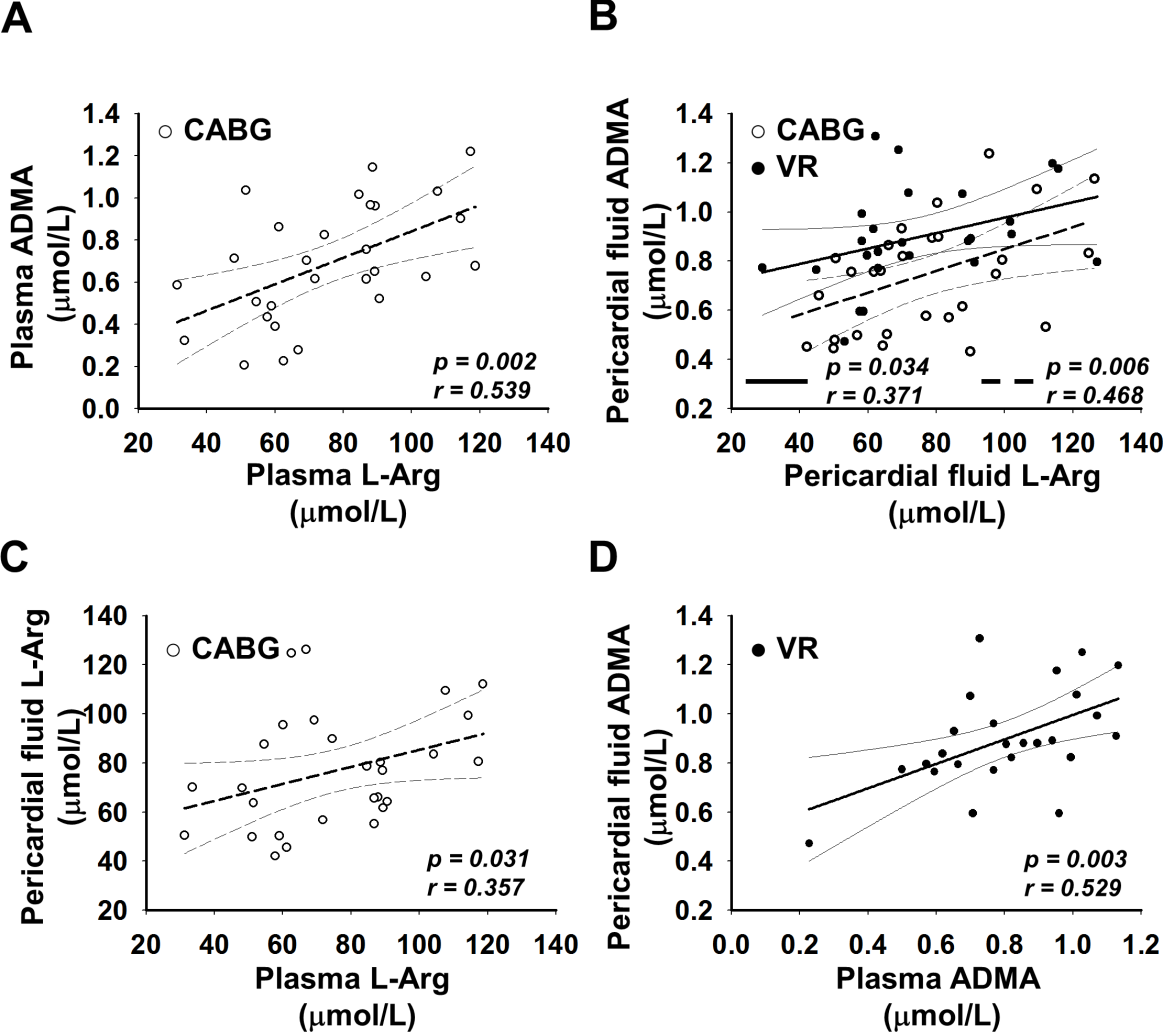
Correlations between the levels of L-Arg and asymmetric dimethylarginine (ADMA) in plasma and pericardial fluid (PF) of patients undergoing coronary artery bypass graft or valve replacement surgery. (A) plasma L-Arg vs. ADMA of CABG patients (*y* = 0.006*x* + 0.21, *r* = 0.539, *p* = 0.002); (B) PF L-Arg vs. ADMA of CABG and VR patients (CABG: *y* = 0.005*x* + 0.39, *r* = 0.468, *p* = 0.006; VR: *y* = 0.003*x* + 0.67, *r* = 0.371, *p* = 0.034); (C) plasma vs. PF L-Arg of CABG patients (*y* = 0.347*x* + 50.69, r = 0.357, p = 0.031); (D) plasma vs. PF ADMA of VR patients (*y* = 0.498*x* + 0.50, r = 0.529, p = 0.003).

In NCP, there was no significant correlation between the levels of L-Arg and ADMA in plasma. However, we found positive significant correlation between levels of pl L-Arg and ADMA in CABG patients (Fig 2A), and between PF L-Arg and ADMA in both CABG and VR patients (Fig 2B). Furthermore, we found correlation between L-Arg levels of plasma and PF in CABG patients (Fig 2C), and ADMA levels of plasma and PF in VR patients (Fig 2D). However, we did not find correlation neither between the pl L-Arg and PF ADMA, nor between the PF L-Arg and pl ADMA in CABG and in VR group, respectively.

### Echocardiographic parameters of CABG and VR patients

**Fig 3:**
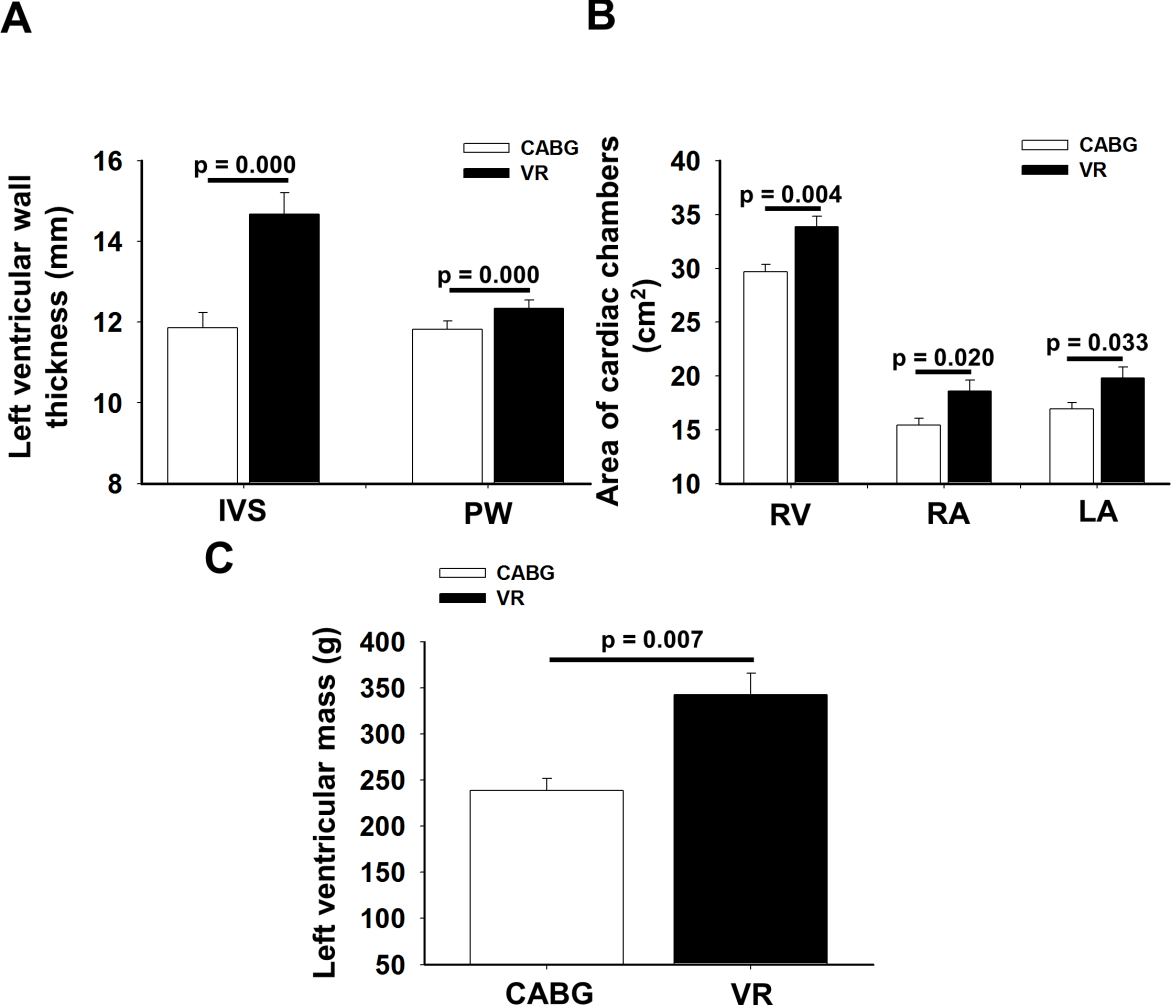
Morphological parameters of ventricles and atria of patients undergoing coronary artery bypass graft (CABG, n = 28) or valve replacement (VR, n = 25) surgery. (A) The thickness of interventricular septum (IVS) and posterior wall (PW), (B) the right ventricular (RV), the right atrial (RA) and the left atrial (LA) areas and (C) the left ventricular mass significantly higher in VR compared to CABG. Mean±SEM. p<0.05.

As Fig 3 demonstrates, the thickness of interventricular septum (IVS), posterior wall of left ventricle (PW) and right ventricular (RV), and right atrial (RA) and left atrial (LA) areas were significantly greater in VR patients than that of CABG patients (IVS: 14.7±0.5 mm vs. 11.8±0.4 mm, p = 0.000; PW: 12.8±0.3 mm vs. 11.5±0.2 mm, p = 0.000; RV: 33.7±1.0 cm^2^ vs. 29.9±0.7 cm^2^, p = 0.004; RA: 18.5±1.1 cm^2^ vs. 15.5±0.7 mm^2^, p = 0.020; LA: 19.6±1.0 cm^2^ vs. 17.1±0.6 cm^2^, p = 0.033). (Fig 3A and 3B). Also, LVM was significantly higher in VR patients compared to CABG patients (318.9±19.3 g vs. 238.1±14.5 g, p = 0.007) (Fig 3C), whereas left ventricular ejection fraction (LVEF) was significantly higher in CABG as compared to VR patients (CABG: 54.6±1.4% vs. VR: 59.5±1.5%, p = 0.05).

### Correlation between the levels of ADMA and echocardiographic parameters

**Fig 4:**
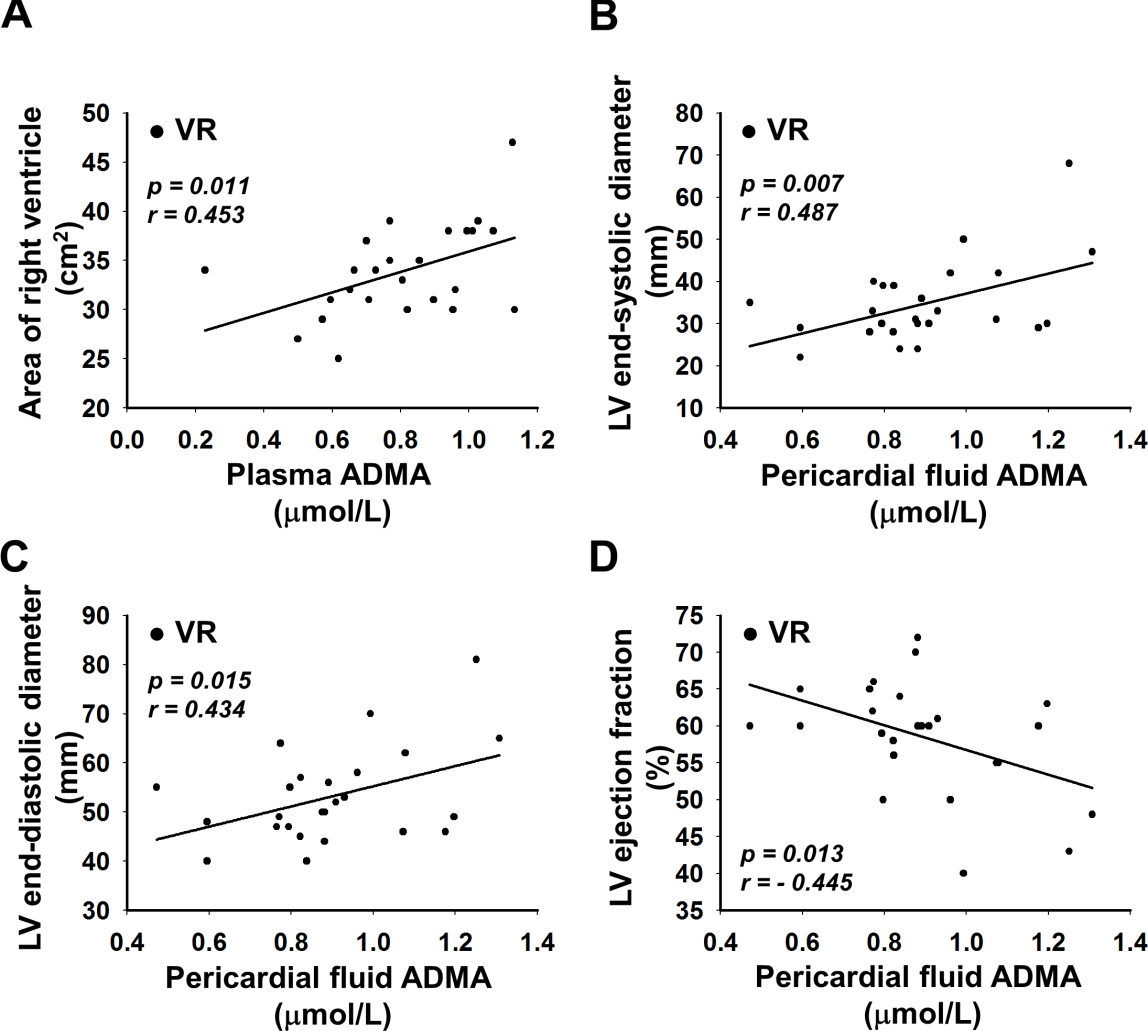
Correlations between the levels of asymmetric dimethylarginine (ADMA) and echocardiographic parameters of patients undergoing valve replacement (VR) surgery. (A) plasma ADMA vs. area of right ventricle (*y* = 10.438*x* + 25.49, *r* = 0.453, *p* = 0.011); (B) PF ADMA vs. left ventricular (LV) end-systolic diameter (*y* = 23.689*x* + 13.53, *r* = 0.487, *p* = 0.007); (C) PF ADMA vs. LV end-diastolic diameter (*y* = 20.531*x* + 34.72, r = 0.434, p = 0.015); D: PF ADMA vs. LV ejection fraction (*y* = -16.779*x* + 73.55, r = -0.445, p = 0.013).

We found positive correlation between the ADMA levels of plasma and RV area (r = 0.453, p = 0.011; Fig 4A), PF ADMA and Ds of LV (r = 0.487, p = 0.007; Fig 4B), and Dd of LV (r = 0.434, p = 0.015; Fig 4C) in VR patients. Furthermore, we found negative correlation between ADMA levels of pericardial fluid and LVEF in VR patients (r = -0.445, p = 0.013; Fig 4D), but not in CABG patients. However, we did not find correlations between ADMA levels of plasma and pericardial fluid with other echocardiographic parameters, neither in CABG nor in VR patients (Table 3).

**Table 3:** Correlations between ADMA levels and echocardiographic parameters of patients undergoing VR surgery.

ADMA levels vs. echocardiographic parameters	R	R^2^	p
Plasma ADMA vs RV	0.453	0.206	0.011
PF ADMA vs RV	0.132	0.017	0.265
Plasma ADMA vs IVS	0.123	0.015	0.279
PF ADMA vs IVS	0.137	0.019	0.257
Plasma ADMA vs PW	-0.114	0.013	0.294
PF ADMA vs PW	0.176	0.031	0.200
Plasma ADMA vs Dd of LV	0.162	0.026	0.220
PF ADMA vs Dd of LV	0.434	0.189	0.015
Plasma ADMA vs Ds of LV	0.163	0.027	0.218
PF ADMA vs Ds of LV	0.487	0.237	0.007
Plasma ADMA vs RA	0.175	0.031	0.201
PF ADMA vs RA	0.050	0.003	0.406
Plasma ADMA vs LA	0.183	0.033	0.191
PF ADMA vs LA	0.104	0.011	0.310
Plasma ADMA vs LVM	-0.018	0.000	0.466
PF ADMA vs LVM	0.201	0.040	0.168
Plasma ADMA vs LVEF	-0.238	0.057	0.126
PF ADMA vs LVEF	-0.445	0.198	0.013

ADMA: asymmetric dimethylarginine; PF: pericardial fluid; VR: valve replacement; RV: area of right ventricle; IVS: thickness of interventricular septum; PW: thickness of posterior wall; Ds of LV: end-systolic diameter of left ventricle; Dd of LV: end-diastolic diameter of left ventricle; RA: area of right atria; LA: area of left atria; LVM: left ventricular mass; LVEF: left ventricular ejection fraction; R: Pearson’s correlation coefficient; R^2^: R-squared value.

The salient findings of the present study are that

- L-Arg and its methylated derivative ADMA are present in the pericardial fluid (PF) of patients undergoing coronary artery bypass graft (CABG) and valve replacement (VR) surgeries,
- in CABG patients, plasma L-Arg concentration was higher compared to that of VR patients, whereas in VR patients, PF ADMA concentration was higher compared to that of CABG patients,
- we have found positive correlation between plasma L-Arg and ADMA levels in CABG patients, between pericardial fluid L-Arg and ADMA levels in both CABG and VR patients, between plasma L-Arg and pericardial fluid L-Arg levels in CABG patients, and between plasma and pericardial fluid ADMA in VR patients,
- the L-Arg/ADMA ratio was smaller in the PF and plasma of VR than in CABG patients,
- we have found positive correlation between plasma ADMA levels and area of right ventricle, between pericardial fluid ADMA levels and end-systolic, and end-diastolic diameter of the left ventricle, and negative correlation between pericardial fluid ADMA levels and left ventricular ejection fraction in VR patients.

### Human PF contains bioactive substances and biomarkers

Previous studies have demonstrated that human PF contains bioactive substances, among them some of which are vasoactive, such as endothelin 1 (ET 1) [5]. Also, it has been reported that the level of these substances varies in different cardiac diseases [9, 38]. Furthermore, it has been revealed that in cardiac patients, certain bioactive substances, such as ET 1 present in higher concentration in PF compared to the plasma [39]. Recently, a false substrate for NOS, ADMA, which is a methylated derivative of L-Arg produced by PRMT1 and degraded by dimethylarginine-dimethylamino-hydrolase (DDAH), has gained attention.

ADMA has been noted as a cardiovascular risk factor due to its increased plasma levels in several cardiovascular diseases [40]. Furthermore, it has been demonstrated that ADMA impairs NO-regulation of vascular tone in part, by direct inhibition of endothelial NO synthase (eNOS) and by reducing bioavailability of NO by increased production of reactive oxygen species (ROS) due to activation of the vascular renin-angiotensin system as shown in isolated arterial vessels in vitro [18–20].

### Human PF contains a high levels of ADMA

In the present study, we found that PF of patients undergoing CABG and VR contains L-Arg and ADMA (Fig 1). There are studies reporting values between 50 and 100 μmol/L for L-Arg, and 0.3–0.8 μmol/L for ADMA in humans [41–43]. The values of the plasma levels of L-Arg, and ADMA of NCP obtained in this study fell into this range. Because, PF of healthy people has not yet been investigated, therefore there are no exact reference values are available for concentrations of L-Arg and ADMA in PF in healthy individuals.

Importantly, the level of ADMA has been found to be elevated in various cardiovascular diseases [31, 44–46]. Also, it has been demonstrated, that plasma level of ADMA changed after stent placement in patients with coronary artery disease (CAD) [47]. Furthermore, Lu et al showed that plasma ADMA level significantly correlated with the severity of CAD [48]. We found that both plasma and PF levels of L-Arg was about 100 fold higher than that of ADMA in both CABG and VR patients. Furthermore, in CABG patients and VR patients we observed that plasma ADMA was near normal or reached the upper limit of the normal range. In VR patients, we observed that the levels of PF ADMA were significantly higher as compared to the CABG patients (Fig 1B).

### L-Arg/ADMA ratio in plasma and PF as an indicator of NO bioavailability

Previous studies have suggested that L-Arg/ADMA ratio reflects NO bioavailability [36, 49]. It is known that a sufficient amount of the substrate, L-Arg is necessary for NOS to produce NO. However, ADMA in pathophysiological relevant concentrations–being a false substrate—is a competitive inhibitor of eNOS, thus inhibits NO formation resulting in reduced NO synthesis [18–20]. It has been shown that the L-Arg/ADMA ratio in plasma is about 100:1 in healthy individuals [34, 50]. Previously, it has been established that low L-Arg/ADMA ratio in acute myocardial infarction (AMI) can be linked to the severity of coronary insufficiency [51]. Also, it was previously reported that there is a correlation between coronary atherosclerotic score and plasma L-Arg/ADMA ratio indicating that changes in this ratio is linked to the severity of CAD [48]. In the present study, we found no significant difference in plasma L-Arg/ADMA ratio between the NCP, and the CABG and VR patients, whereas both the plasma and PF L-Arg/ADMA ratios showed a significant difference between the CABG and VR patients (Fig 1C).

We have found that both in CABG and VR patients eGFR was similar, <60ml/min/1.73 m^2^ (Table 1), suggesting the presence of renal insufficiency (because it has been defined as GFR < 75 ml/min/1.73 m^2^) which is known to be strongly associated with heart diseases [52, 53]. Also, we found a significant inverse correlation between plasma L-Arg and eGFR in the CABG group, which is may be due to the higher L-Arg levels in the plasma of CABG patients, which could be due to the higher L-Arg levels in CABG patients [24]. Previous studies have shown that reduced L-Arg/ADMA ratio is associated with reduced GFR in patients with CKD [36] and that the kidneys releases L-Arg into the plasma, which may increase plasma level L-Arg [54]. However, we have found no significant correlation between L-Arg/ADMA ratio and eGFR of VR and CABG patients, suggesting that the primary reason for this is not related to the kidney dysfunction. Different levels of ADMA in CABG and VR patients indicate that ADMA may have specific roles in the pathomechanisms of cardiac events and reflects various pathological events of the heart. Taken together, these suggest that ADMA may reflect the pathophysiological and-morphological changes of the myocardium.

### ADMA in PF and left ventricular remodeling/hypertrophy

Left ventricular hypertrophy is the result of interaction between a chronic hemodynamic overload and non-hemodynamic factors [55]. The diastolic dysfunction of the heart is known to be the first predictor of left ventricular failure [56]. In the present study, the majority of VR patients suffered from aortic stenosis, which caused significant chronic pressure overload of the left ventricle [57]. We found, that echocardiographic parameters, which are characteristics of left ventricular hypertrophy, such as thickness of IVS, and parameter of LVM increased significantly in VR patients compared to CABG patients (Fig 3A and 3C). In the VR patients, areas of LA, RA and parameter of RV area exhibited significant increase in comparison of CABG patients (Fig 3B).

The role of NO in the development of hypertrophy and remodeling of the cardiac muscle in response to chronic changes in mechanical constraints (i.e., volume or pressure overload) is important, because altered morphology of the heart affects contractile performance [13]. There are several lines of evidence presented in previous decades suggesting that presence of adequate level of NO limits the hypertrophic growth of the myocardium [15]. One of the mechanisms that may explain the association between ADMA and cardiovascular disease is the ADMA-induced cardiac hypertrophy. Several alternative mechanisms have also been proposed to explain the association between ADMA and cardiac hypertrophy [58]. In cardiac myocytes in cell culture, it has been demonstrated that ADMA can activate fibroblast growth factors receptors. This can lead to myocardial hypertrophy and fibrosis, or induce excessive local activation of the renin-angiotensin mechanism [59].

**Fig 5:**
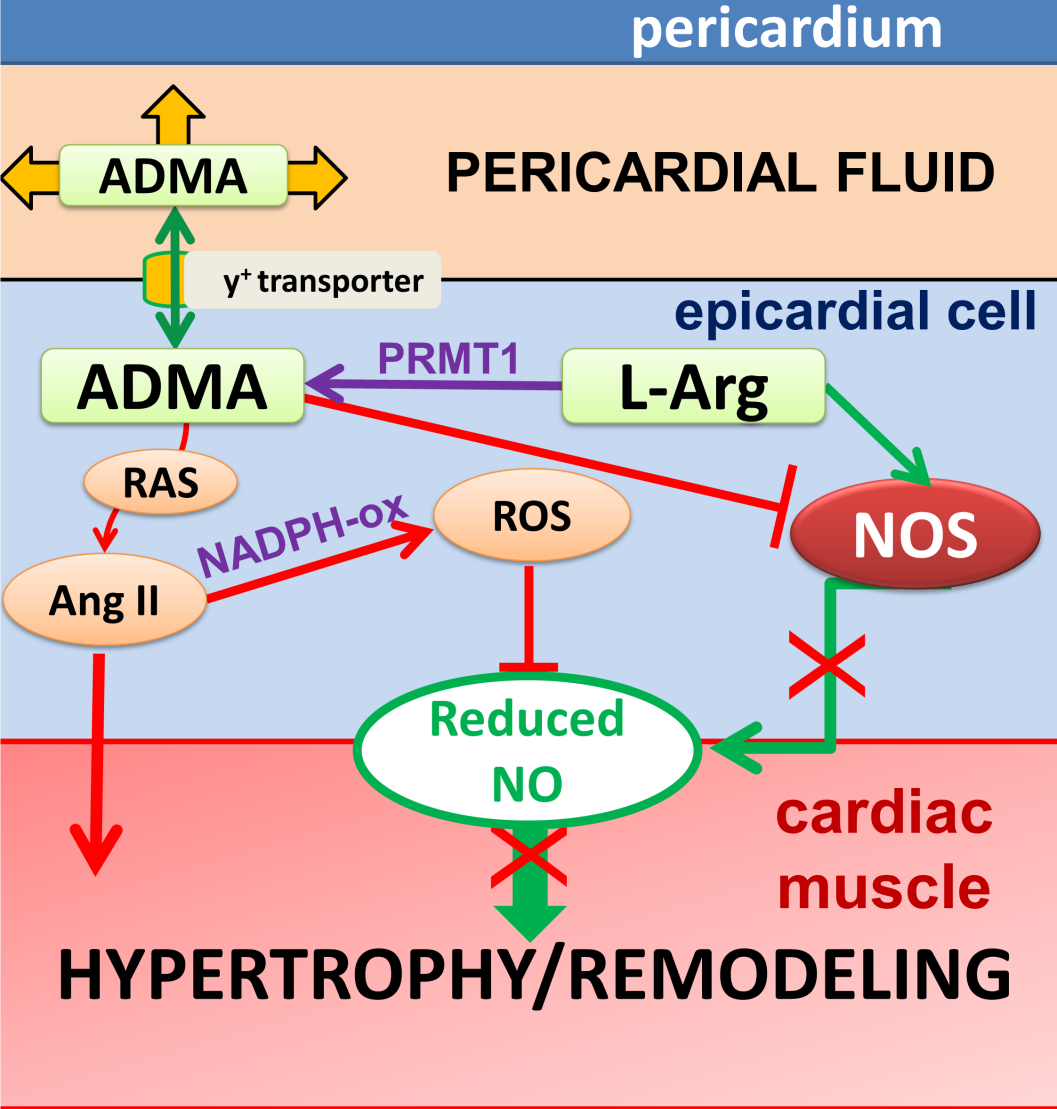
Proposed mechanisms by which elevated level of ADMA in pericardial fluid elicits hypertrophy/remodeling of cardiac muscle: accordingly, reduced NO bioavailability and increased level of Ang II together leads to development of cardiac hypertrophy/remodeling. ADMA–asymmetric dimethylarginine, NO–nitric oxide, NOS–endothelial NO synthase, RAS–renin-angiotensin-system, Ang II–angiotensin II, ROS–reactive oxygen species.

Normal level of NO and activity of NOS are essential for the prevention of heart remodeling, therefore decreased NO availability may lead to a loss of such protection. In Fig 5, we have summarized the potential mechanism of action of ADMA in modulation cardiac morphology. We recently proposed a potential mechanism by which increased serum ADMA reduces the bioavailability of NO [47]. Previously we have also shown that elevated levels of ADMA can activate the renin-angiotensin system in the arteriolar wall. This can elicit increased production of Ang II, which then activates NAD(P)H oxidase leading to increased levels of reactive oxygen species interfering with the bioavailability of NO [19]. The activation of RAS increases the level of Ang II, which is known to be a growth hormone [60]. These observations are in concordance with previous studies and suggest that reduced level of NO [16] and increase activation of local RAS [61] together promote cardiac hypertrophy (Fig 4). Elevated level of ADMA in the pericardial fluid of patients in the VR patients correlates with left ventricular remodeling/hypertrophy and thus it can serve as a biomarker (Fig 5).

### ADMA in the pericardial fluid

PF has been considered as a passive ultrafiltrate of the blood plasma resulting by hydrostatic pressure difference between the plasma and PF and osmotic concentration gradient, as well [3]. It has also been shown that some substances in PF are derived from the cardiac interstitium, such as adenine nucleosides [6] and other cardiac biomarkers [62]. In the present study, we found that L-Arg levels both in the plasma and pericardial fluid obtained from CABG and VR patients were in the normal range (Fig 1A), whereas, ADMA levels reached the maximum of the normal range in these patients (Fig 1B).

The positive correlation between plasma L-Arg and ADMA in CABG patients (Fig 2A) suggests that pericardial ADMA may originate from the plasma. However, as the slope of the curve shows, pericardial ADMA may originate not only from the plasma, but also from cardiac tissues. Both in CABG and in VR patients, we observed significant positive correlations between L-Arg and ADMA in pericardial fluid (Fig 2), suggesting that ADMA can be formed in PF from L-Arg, but in different compartments, as slopes of the lines are different. Taken together, these suggest that pericardial fluid ADMA in part origins from cardiac tissues and more amount of ADMA is formed in VR than in CABG patients. Interestingly, in VR patients we did not find correlation between plasma L-Arg and plasma ADMA, and between plasma L-Arg and pericardial fluid ADMA. In our interpretation, these suggest that in VR patients ADMA may be generated in cardiac tissues from L-Arg, not just from plasma L-Arg. Although, the positive correlation between pericardial L-Arg and ADMA (Fig 2B), and between plasma and pericardial fluid ADMA suggest that in PF ADMA is metabolized from the pericardial fluid L-Arg, however this correlation is not proportional. This latter findings and the positive correlation between plasma and pericardial fluid ADMA (Fig 4D) indicate that ADMA–which is produced and eliminated by several similar metabolic pathways in PF and plasma—may diffuses between the two compartments.

## Conclusions

In conclusion, based on present and previous findings, we suggest that elevated levels of asymmetric dimethyl-arginine (ADMA) in the pericardial fluid of cardiac patients could indicate important pathophysiological mechanisms, such as absolute or relative cardiac ischemia and hypoxia leading to reduced bioavailability of nitric oxide, which–together with the locally released growth hormone Ang II—can contribute to the development of cardiac hypertrophy and remodeling (Fig 5). We propose that analyzing of pericardial fluid could be a valuable diagnostic tool, whereas interfering with the contents and effects of pericardial fluid open up new therapeutic options to beneficially modify cardiac function and structure.
